# Prognostic Role of *TMED3* in Clear Cell Renal Cell Carcinoma: A Retrospective Multi-Cohort Analysis

**DOI:** 10.3389/fgene.2019.00355

**Published:** 2019-04-17

**Authors:** Mihyang Ha, Hwan Moon, Dongwook Choi, Wonmo Kang, Ji-Hong Kim, Keon Jin Lee, Dongsu Park, Chi-Dug Kang, Sae-Ock Oh, Myoung-Eun Han, Yun Hak Kim, Dongjun Lee

**Affiliations:** ^1^Department of Anatomy, School of Medicine, Pusan National University, Yangsan, South Korea; ^2^Department of Premedicine, School of Medicine, Pusan National University, Yangsan, South Korea; ^3^Division of Drug Process Development, New Drug Development Center, Osong Medical Innovation Foundation, Cheongju, South Korea; ^4^Department of Molecular and Human Genetics, Baylor College of Medicine, Houston, TX, United States; ^5^Department of Pathology and Immunology, Baylor College of Medicine, Houston, TX, United States; ^6^Center for Skeletal Medicine and Biology, Baylor College of Medicine, Houston, TX, United States; ^7^Department of Biochemistry, Pusan National University School of Medicine, Yangsan, South Korea; ^8^Department of Convergence Medical Science, Pusan National University School of Medicine, Yangsan, South Korea; ^9^Department of Anatomy, Biomedical Research Institute, School of Medicine, Pusan National University, Yangsan, South Korea; ^10^Department of Biomedical Informatics, Biomedical Research Institute, School of Medicine, Pusan National University, Yangsan, South Korea

**Keywords:** TMED3, TCGA, ICGC, clear cell renal cell carcinoma, prognosis

## Abstract

Transmembrane p24 trafficking protein 3 (TMED3) is a metastatic suppressor in colon cancer and hepatocellular carcinoma. However, its function in the progression of clear cell renal cell carcinoma (ccRCC) is unknown. Here, we report that TMED3 could be a new prognostic marker for ccRCC. Patient data were extracted from cohorts in the Cancer Genome Atlas (TCGA) and the International Cancer Genome Consortium (ICGC). Differential expression of TMED3 was observed between the low stage (Stage I and II) and high stage (Stage III and IV) patients in the TCGA and ICGC cohorts and between the low grade (Grade I and II) and high grade (Grade III and IV) patients in the TCGA cohort. Further, we evaluated *TMED3* expression as a prognostic gene using Kaplan-Meier survival analysis, multivariate analysis, the time-dependent area under the curve (AUC) of Uno’s C-index, and the AUC of the receiver operating characteristics at 5 years. The Kaplan-Meier analysis revealed that *TMED3* overexpression was associated with poor prognosis for ccRCC patients. Analysis of the C-indices and area under the receiver operating characteristic curve further supported this. Multivariate analysis confirmed the prognostic significance of *TMED3* expression levels (*P* = 0.005 and 0.006 for TCGA and ICGC, respectively). Taken together, these findings demonstrate that TMED3 is a potential prognostic factor for ccRCC.

## Introduction

The transmembrane emp24 domain (TMED) protein family is involved in the vesicular trafficking of proteins and innate immune signaling (Strating and Martens, 2009; Zheng et al., 2016). TMED proteins contain a Golgi dynamics domain and function in Golgi dynamics and intracellular protein trafficking (Jenne et al., 2002; Carney and Bowen, 2004; Luo et al., 2007;Jerome-Majewska et al., 2010). Recent studies have implicated TMED7 in the regulation of TLR4 signaling (Palsson-McDermott et al., 2009; Doyle et al., 2012; Liaunardy-Jopeace et al., 2014), and TMED1 is involved in the ST2L-IL33 axis (Connolly et al., 2013). In addition, a recent study showed that TMED3 overexpression was significantly correlated with an aggressive phenotype of HCC and poor prognosis (Zheng et al., 2016). In HCC, TMED3 promotes metastasis through IL-11/STAT3 signaling. However, the clinical significance of TMED3 and its role in other malignancies are unknown.

Kidney cancer is among the top 10 cancers, and 30% of patients with kidney cancer present with metastatic disease (Nickerson et al., 2008). Renal cell carcinoma (RCC) accounts for 90% of kidney cancers (Choueiri and Motzer, 2017; Siegel et al., 2018), and clear cell renal cell carcinoma (ccRCC) is the most common type of kidney cancer (Srigley et al., 2013). However, 30% of patients with ccRCC have been diagnosed with advanced cancer (Karakiewicz et al., 2007), and the therapeutics available for renal cancer is not very effective. Therefore, there is a great need for new drugs and biomarkers for ccRCC.

Thus far, the prognostic significance of TMED3 in ccRCC is unknown. In this study, we present the first data on *TMED3* expression in ccRCC in a well-defined cohort from the TGCA (Cerami et al., 2012; Cancer Genome Atlas Research Network et al., 2013) and ICGC (International Cancer Genome Consortium et al., 2010) primary ccRCC cohorts. The statistical analysis suggested that TMED3 could be a useful prognostic factor in ccRCC.

## Materials and Methods

### Patient Data Acquisition and Statistical Analysis

The data were downloaded from TCGA (Cerami et al., 2012; Cancer Genome Atlas Research Network et al., 2013) and ICGC (International Cancer Genome Consortium et al., 2010) from the ICGC data portal ^1^ in March 2018. We downloaded mRNA expression (TCGA, RSEM normalization; ICGC, RPKM normalization) and clinical information. Samples with insufficient information (gene expression values and survival information) were excluded from the analysis. GSE11024 (Affymetrix U133 Plus 2.0 Array) (Kort et al., 2008), GSE12606 (Affymetrix U133 Plus 2.0 Array) (Stickel et al., 2009), and GSE14762 (Affymetrix U133 Plus 2.0 Array) (Wang et al., 2009) were downloaded from GEO database using “GEOquery” R package. In the stage-related analysis, only the “Not Available (NA)” value of the stage was excluded. When the grade-related analysis was performed, only the “NA” value of the grade was excluded. These analyses were performed using R software version 3.5.0 (R Core Team, 2018).

To identify the differences of *TMED3* expression values between low stages (I and II) and high stages (III and IV), we performed Wilcoxon rank sum test using “coin” R package because the differences were not a normal distribution. We used Kruskal–Wallis test with Bonferroni adjustment to identify the differential expression of *TMED3* in different T stages using appropriate statistical methods (GSE11024 and GSE14762, Welch two sample *t*-test; GSE12606, paired *t*-test). Survival analyses were performed to predict overall survival (OS). We used three methods, (1) Uno’s C-index in a time-dependent Area Under the Curve (AUC) analysis, (2) AUC values in receiver operating characteristics (ROC) at 5 years, and (3) Kaplan-Meier survival analysis, to evaluate the accuracy of the discrimination, as described previously (Cho et al., 2018; Han et al., 2018). These values were obtained using the R packages “survival” and “survAUC.” The C-index is a global measure of the fitness of a survival model for continuous event time in clinical studies (Uno et al., 2011; Kim et al., 2017a,b). In the Kaplan-Meier analyses, we determined the optimal cutoff value that had the maximal Uno’s C-index by fivefold cross-validation (Table 2). We then used univariate and multivariate Cox regression to compare the effect of *TMED3* expression level as a categorical value on prognosis, along with other clinical variables. In the multivariate analysis with the stepwise selection, we included clinical factors that were not associated with survival in the univariate analysis. All statistical analyses were performed using R.

## Results

### Overexpression of *TMED3*

**FIGURE 1:**
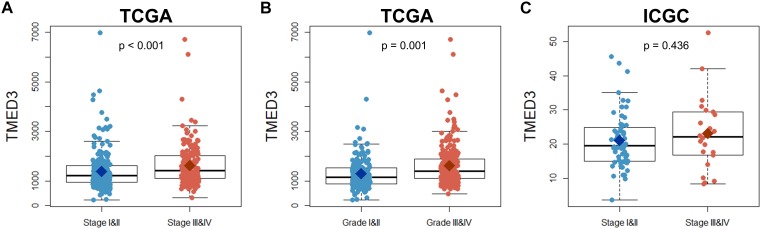
Comparison of *TMED3* gene expression between low (Stage I and II) and high stage (Stage III and IV) patients in the TCGA and ICGC ccRCC cohorts as well as the low (Grade I and II) and high grade (Grade III and IV) patients in the TCGA ccRCC cohort. **(A,B)** *TMED3* expression levels in ccRCC patients from the TCGA cohort. **(C)** *TMED3* expression levels in ccRCC cases from the ICGC cohort.

The study included 446 patients from the TCGA and 91 patients from the ICGC (Hoshida et al., 2009; International Cancer Genome Consortium et al., 2010; Cerami et al., 2012; Cancer Genome Atlas Research Network et al., 2013; Shtraizent et al., 2017). Patient information that was used in the current study is shown in Table 1. The expression of *TMED3* was compared between low (Stage I and II) and high stage (Stage III and IV) ccRCC patients in the TCGA and ICGC cohorts, and between low (Grade I and II) and high grade (Grade III and IV) ccRCC patients from the TCGA cohort. The *TMED3* expression levels in the high stage and grade cohorts were much higher than in the low stage and grade cohorts (Figure 1). The groups with statistically significant *TMED3* differences were only two groups (T1 vs. T2 and T1 vs. T3 in TCGA) (Supplementary Figure S1). Additionally, we confirmed *TMED3* expression in cancer tissues are higher than normal tissues by using GSE11024, GSE12606, and GSE14762) (Supplementary Figure S2).

**Table 1:** Information on patients included in this study.

Age (mean ± standard deviation)		TCGA 60.62 ± 12.80	ICGC 60.47 ± 10.03
Gender	Male	290	52
	Female	156	39
T stage	T1	221	54
	T2	57	13
	T3	161	22
	T4	7	2
N stage	N0	205	79
	N1	14	2
M stage	M0	376	81
	M1	70	9
TNM stage	I	216	48
	II	46	12
	III	111	13
	IV	71	9
	NA	2	9
Grade	I	9	–
	II	189	–
	III	175	–
	IV	68	–
	NA	5	–
Total patients		446	91

**Table 2:** *TMED3* expression levels in the TCGA and ICGC cohorts.

		TCGA	ICGC
*TMED3*	Median	1299.2	19.921
	Mean	1478.6	21.891
	Cutoff	1360.708	23.942

### Prognostic Value of *TMED3* Expression in ccRCC Patients

**FIGURE 2:**
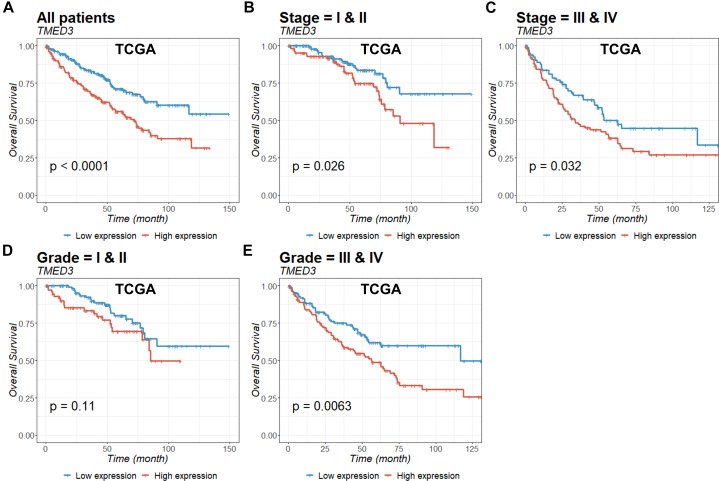
Kaplan-Meier survival curves of ccRCC patients according to *TMED3* expression levels. Overall survival of all **(A)**, stage I and II **(B)**, stage III and IV **(C)**, grade I and II **(D)**, and grade III and IV **(E)** patients in the TCGA cohort.

**FIGURE 3:**
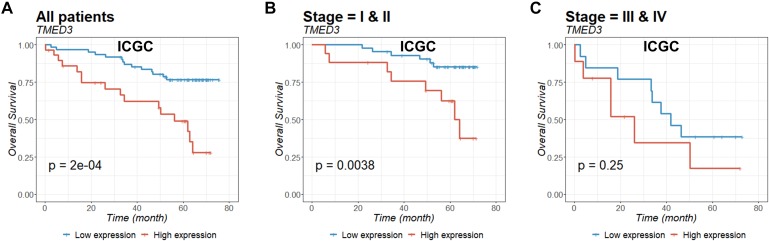
Overall survival of all **(A)**, stage I and II **(B)**, and stage III and IV **(C)** patients in the ICGC cohort were examined according to *TMED3* gene expression levels. *P*-values were calculated by the log-rank test and are shown at the bottom left of each panel.

To evaluate the prognostic value of *TMED3* in ccRCC, we analyzed Kaplan-Meier survival curves for *TMED3* gene expression and survival from the TCGA (Figure 2) and ICGC (Figure 3) cohorts. The high *TMED3* expression group had a significantly shorter survival than the low *TMED3* expression group in the TCGA (Figure 2) and ICGC cohorts (Figure 3). The prognostic value was further confirmed using multivariate analysis (*P* = 0.005 and 0.006 for the TCGA and ICGC cohorts, respectively; Table 3).

**Table 3:** Univariate and multivariate analysis of overall survival in each cohort.

Variables	Univariate cox regression				Multivariate cox regression (stepwise method)			
	*P*-value	Hazard ratio	95% confidence interval		*P*-value	Hazard ratio	95% confidence interval	
**TCGA**								
TMED3	<0.001^∗∗∗^	1.927	1.388	2.674	0.036^∗^	1.614	1.031	2.526
Age	<0.001^∗∗∗^	1.033	1.018	1.047	0.002^∗∗^	1.034	1.012	1.055
Gender	0.333	0.850	0.612	1.181	–	–	–	–
T stage (I, II vs. III, IV)	<0.001^∗∗∗^	2.912	2.101	4.035	0.002^∗∗^	2.103	1.303	3.396
N stage (0 vs. 1)	0.0011^∗∗^	3.215	1.599	6.464	–	–	–	–
M stage (0 vs. 1)	<0.001^∗∗∗^	4.189	3.005	5.838	<0.001^∗∗∗^	3.371	2.021	5.623
**ICGC**								
TMED3	<0.001^∗∗∗^	3.612	1.756	7.429	<0.001^∗∗∗^	3.543	1.718	7.306
Age	0.109	1.031	0.993	1.071	–	–	–	–
Gender	0.863	1.066	0.517	2.194	–	–	–	–
T stage (I, II vs. III, IV)	<0.001^∗∗∗^	3.786	1.838	7.801	<0.001^∗∗∗^	4.165	2.011	8.628
N stage (0 vs. 1)	0.444	2.184	0.295	16.190	–	–	–	–
M stage (0 vs. 1)	<0.001^∗∗∗^	8.305	3.615	19.080	–	–	–	–

*^∗^, ^∗∗^, and ^∗∗∗^indicate significance (<0.05, <0.01, and <0.001).*

**FIGURE 4:**
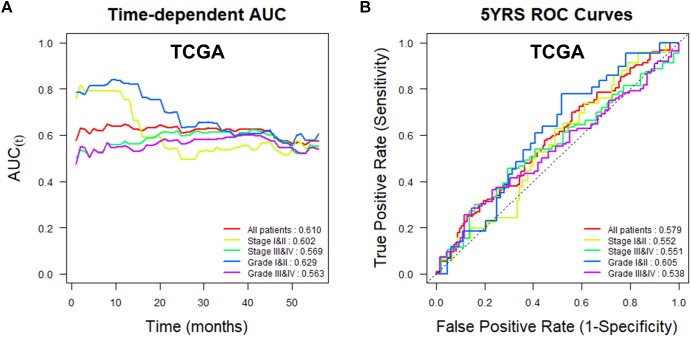
Time-dependent area under the curve (AUC) and receiver operating characteristic (ROC) curves at 5 years according to *TMED3* expression levels in the TCGA cohort. **(A)** Time-dependent AUC and **(B)** ROC curves at 5 years for patients in the TCGA cohort according to *TMED3* expression levels. C-index values are shown at the bottom right in **(A)**. AUC values at 5 years are shown at the bottom right in **(B)**.

**FIGURE 5:**
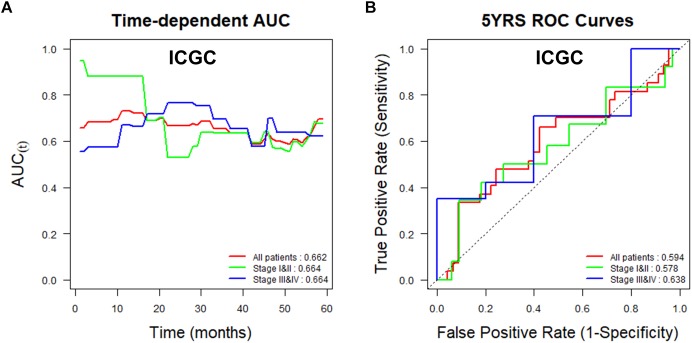
Time-dependent area under the curve (AUC) and receiver operating characteristic (ROC) curves at 5 years according to *TMED3* expression levels in the ICGC cohort. **(A)** Time-dependent AUC and **(B)** ROC curves at 5 years for patients in the ICGC cohort according to *TMED3* expression levels. C-index values are shown at the bottom right in **(A)**. AUC values at 5 years are shown at the bottom right in **(B)**.

To assess the utility of *TMED3* expression as a biomarker for ccRCC, we examined Uno’s C-index in a time-dependent AUC analysis and the AUC values for ROCs at 5 years for the TCGA (Figure 4) and ICGC cohorts (Figure 5). *TMED3* had high C-index values in the two independent cohorts (TCGA: 0.610 and ICGC: 0.602; Figure 4A, 5A, respectively). The 5-year ROC graphs also showed high AUC values for the TCGA and ICGC cohorts (TCGA: 0.579 and ICGC: 0.594; Figure 4B, 5B, respectively).

## Discussion

The main purpose of our study is to strengthen the foundation of precision medicine by analyzing big genome data. There is a growing need to find novel prognostic genes for ccRCC. We analyzed the *TMED3* gene from two large independent cohorts as prognostic markers for ccRCC. In the present study, we confirmed that the *TMED3* gene fulfills a sufficient role as a universal prognostic marker for ccRCC. From survival analysis, we found a very good marker (*TMED3*) to predict the prognosis of renal cell carcinoma patients.

*TMED3* showed good predictive power in patients with low- and high-stage ccRCC, and low- and high-grade disease in the TCGA cohort and in patients with low- and high-stage cancer in the ICGC cohort (Figure 4, 5). In addition, *TMED3* overexpression is associated with poor prognosis of ccRCC. A recent study showed that *TMED3* is overexpressed in HCC and that *TMED3* promotes HCC metastasis through IL-11/STAT3 signaling (Zheng et al., 2016). Moreover, STAT3 activation is correlated with *TMED3* expression in HCC. Further, *TMED3* may contribute to the progression of colon cancer (Duquet et al., 2014).

The current treatments for advanced ccRCC are VEGF, VEGFR and mammalian target of rapamycin (mTOR) (Wang et al., 2018) – targeted therapy, but surgical treatment remains the most effective clinical therapy for ccRCC. The ccRCC can easily invade local tissues and metastasize (Yan et al., 2009). In addition, patients with RCC typically respond poorly to radiation and conventional chemotherapy (Linehan, 2012) and ccRCC cells are unsatisfactory and resistant to currently available therapeutics. Further, the rates of recurrence and metastasis for ccRCC remain high due to long-term interactions with the microenvironment (Subramanian and Haas, 2018; Wang et al., 2018). Understanding the mechanisms underlying ccRCC pathogenesis will support the development of more effective therapeutic strategies, including new drugs and biomarkers. With recent advances in biotechnology, the field of bioinformatics has developed rapidly and more potential biomarkers have been discovered (Guan et al., 2018). There are a number of free databases available to the public, including GEO and TCGA databases that contain extensive gene expression data useful to finding heretofore unknown biomarkers and provide a wealth of information that can be used to identify biomarkers (Gao et al., 2018). These new molecular markers can be used in combination with the current staging systems.

Based on our findings in both cohorts, the higher the *TMED3* expression level, the worse the patient prognosis. Although there are limitations in transcriptome-based studies of *TMED3*, we believe that our results are sufficient to suggest the possibility of *TMED3* as a new prognostic biomarker for ccRCC.

## Author Contributions

YK, DL, M-EH, and S-OO contributed to conception and design of the study. DC, WK, J-HK, KL, DP, and C-DK acquired the data. MH and HM analyzed and interpreted the data. DL and YK wrote and reviewed the manuscript. YK supervised the study. All authors read and approved the final version of this manuscript.

## Conflict of Interest Statement

The authors declare that the research was conducted in the absence of any commercial or financial relationships that could be construed as a potential conflict of interest.

## Footnotes

1 dcc.icgc.org

## Supplementary Material

FIGURE S1 Comparison of *TMED3* gene expression according to T stages in the TCGA **(A)** and ICGC **(B)** ccRCC cohorts.

FIGURE S2 Comparison of TMED3 gene expression between cancer and normal kidney in GSE11024 **(A)**, GSE12606 **(B)**, and GSE14762 **(C)**.
