# Supplementary figures and images for: Prognostic Role of TMED3 in Clear Cell Renal Cell Carcinoma: A Retrospective Multi-Cohort Analysis
Source: Front Genet. 2019 Apr 17;10:355. doi: 10.3389/fgene.2019.00355 (PMC6478656; doi:10.3389/fgene.2019.00355)

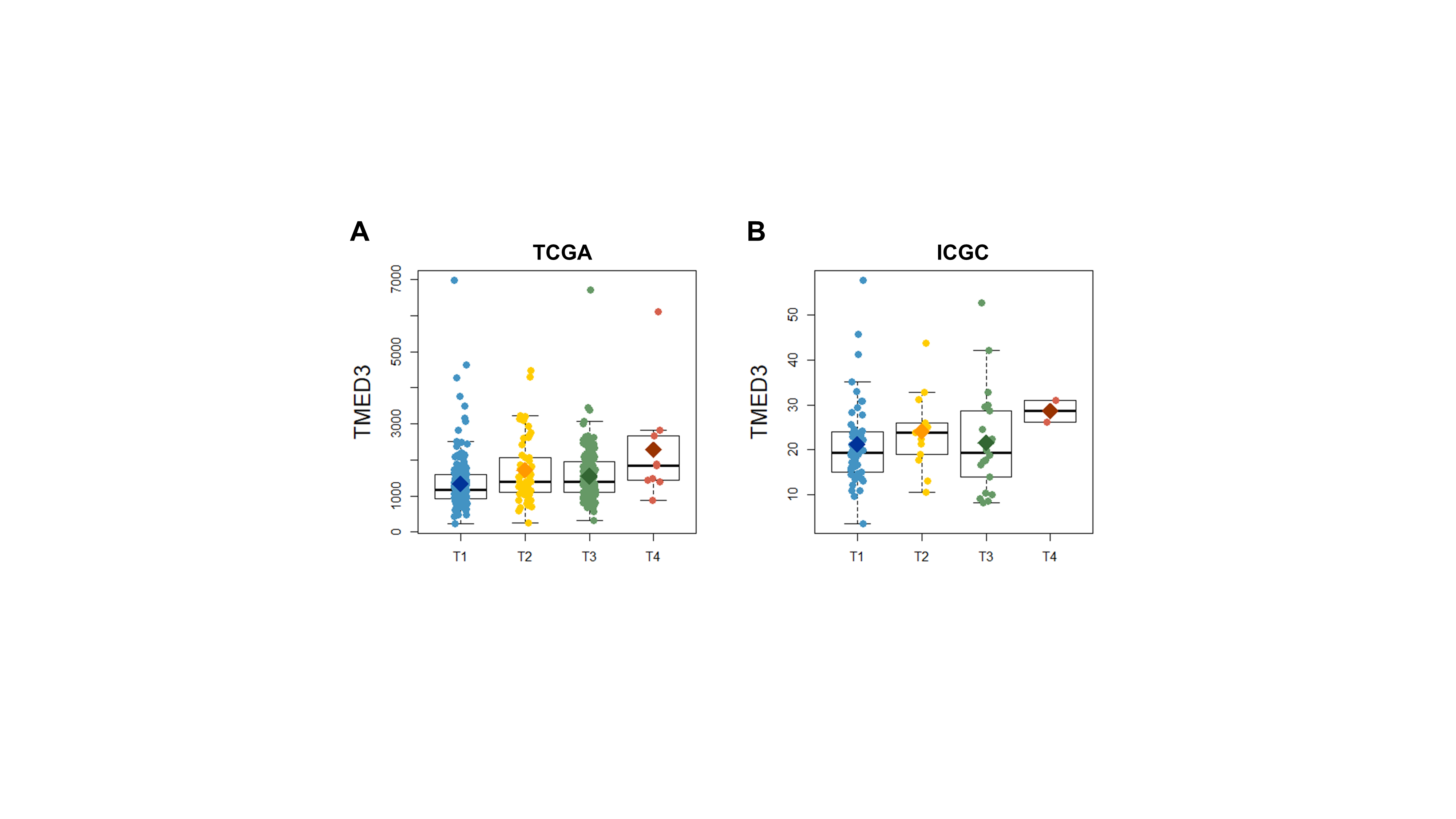

Supplement: FIGURE S1 — Comparison of TMED3 gene expression according to T stages in the TCGA (A) and ICGC (B) ccRCC cohorts. [file Image_1.TIF]

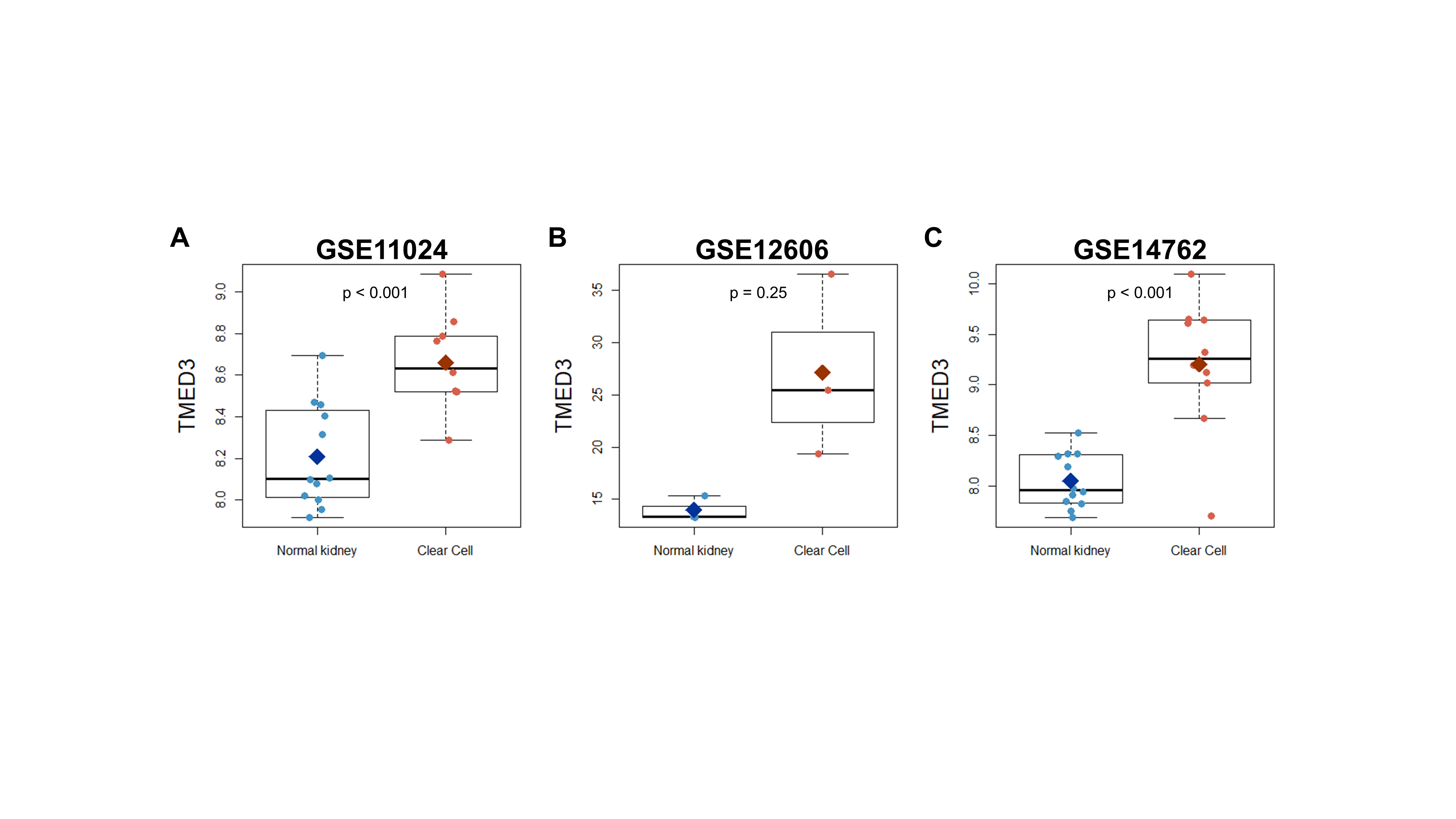

Supplement: FIGURE S2 — Comparison of TMED3 gene expression between cancer and normal kidney in GSE11024 (A), GSE12606 (B), and GSE14762 (C). [file Image_2.TIF]
